# In Situ Polymerization Permeated Three‐Dimensional Li^+^‐Percolated Porous Oxide Ceramic Framework Boosting All Solid‐State Lithium Metal Battery

**DOI:** 10.1002/advs.202003887

**Published:** 2021-03-03

**Authors:** Yiyuan Yan, Jiangwei Ju, Shanmu Dong, Yantao Wang, Lang Huang, Longfei Cui, Feng Jiang, Qinglei Wang, Yanfen Zhang, Guanglei Cui

**Affiliations:** ^1^ Qingdao Industrial Energy Storage Research Institute Qingdao Institute of Bioenergy and Bioprocess Technology Chinese Academy of Sciences Qingdao 266101 P. R. China

**Keywords:** composite electrolyte, flexible–rigid coupling solid electrolyte, in situ polymerization, porous framework, solid‐state lithium battery

## Abstract

Solid‐state lithium battery promises highly safe electrochemical energy storage. Conductivity of solid electrolyte and compatibility of electrolyte/electrode interface are two keys to dominate the electrochemical performance of all solid‐state battery. By in situ polymerizing poly(ethylene glycol) methyl ether acrylate within self‐supported three‐dimensional porous Li_1.3_Al_0.3_Ti_1.7_(PO_4_)_3_ framework, the as‐assembled solid‐state battery employing 4.5 V LiNi_0.8_Mn_0.1_Co_0.1_O_2_ cathode and Li metal anode demonstrates a high Coulombic efficiency exceeding 99% at room temperature. Solid‐state nuclear magnetic resonance results reveal that Li^+^ migrates fast along the continuous Li_1.3_Al_0.3_Ti_1.7_(PO_4_)_3_ phase and Li_1.3_Al_0.3_Ti_1.7_(PO_4_)_3_/polymer interfacial phase to generate a fantastic conductivity of 2.0 × 10^−4^ S cm^−1^ at room temperature, which is 56 times higher than that of pristine poly(ethylene glycol) methyl ether acrylate. Meanwhile, the in situ polymerized poly(ethylene glycol) methyl ether acrylate can not only integrate the loose interfacial contact but also protect Li_1.3_Al_0.3_Ti_1.7_(PO_4_)_3_ from being reduced by lithium metal. As a consequence of the compatible solid‐solid contact, the interfacial resistance decreases significantly by a factor of 40 times, resolving the notorious interfacial issue effectively. The integrated strategy proposed by this work can thereby guide both the preparation of highly conductive solid electrolyte and compatible interface design to boost practical high energy density all solid‐state lithium metal battery.

## Graphical abstract

The poor room‐temperature ionic conductivity of solid electrolyte and incompatibility of solid–solid electrolyte/electrode interface are resolved simultaneously by in situ polymerizing electrode compatible polymer within a highly conductive, three‐dimensional self‐supported porous oxide framework. The composite solid electrolyte guided by this strategy is exploited to achieve excellent cycling performance for high‐voltage solid‐state lithium battery.

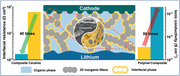

## 1 Introduction

Lithium‐ion battery has been regarded as the most promising next‐generation rechargeable energy storage device in multiple aspects like automobiles, grids, and electric vehicles. Whereas, the safety‐related hazards, which enlist both thermal and chemical threats, arising from the highly combustible organic solvents employed in liquid electrolytes, significantly hinder further development of lithium‐ion battery.^[^ ^1^, ^2^, ^3^ ^]^ Solidifying the liquids to manufacture solid‐state lithium battery (SSLB) has been acknowledged to be a very promising strategy for tackling these safety related issues.^[^ ^4^, ^5^ ^]^

Since its first report in 1970s, poly(ethylene oxide) (PEO), the most widely used solid polymer electrolyte (SPE), has attracted extensive attention due to its superior flexibility and easy manufacturing.^[^ ^6^, ^7^ ^]^ However, the solid polymer usually possesses very low ionic conductivity of 10^−6^ to 10^−8^ S cm^−1^, which thus is very challenging to activate SSLB at ambient temperature.^[^ ^8^ ^]^ One widespread method to enhance the ionic conductivity is the addition of plasticizer into SPE.^[^ ^9^ ^]^ If so, SPE would disable its mechanical properties severely and thus fail to suppress the lithium dendrite growth, which raise the following critical safety issues, e.g., battery short‐circuiting. To tackle the hazard, rigid–flexible coupling method has been demonstrated as a valid strategy, where a separator like thermoset nonwoven plays the role of rigid skeleton to increase the mechanical properties while the ionic conducting polymer guarantees the transport of Li^+^.^[^ ^10^ ^]^ Yet, the rigid skeleton, usually nonconductive nonwovens, does nothing to improve the ionic conductivity of the rigid–flexible coupling electrolyte. To this end, replacing the insulative rigid skeleton by a conductive one is a matter of great urgency to prepare a solid electrolyte with comprehensive performance. Solid inorganic electrolyte (SIE), for example, inorganic oxide of Li_7_La_3_Zr_2_O_12_ (LLZO), Li_1.3_Al_0.3_Ti_1.7_(PO_4_)_3_ (LATP), and sulfide of Li_10_GeP_2_S_12_, possesses relatively higher ionic conductivity of 10^−4^ − 10^−2^ S cm^−1^ at room temperature.^[^ ^11^, ^12^, ^13^, ^14^ ^]^ Consequently, a self‐supported porous SIE framework which can endow both high ionic conductivity and superior mechanical strength is a very promising candidate for the rigid skeleton in rigid–flexible coupling electrolyte. However, there is rare reports on the application of self‐supported porous SIE framework for the rigid–flexible coupling composite electrolyte.

Regardless of the comprehensive performance of the rigid–flexible coupling electrolyte, the incompatible solid‐solid interface of the electrolyte/electrode or within the composite electrode, e.g., the loose interfacial contact or chemical reactions, greatly tortures the performance of SSLB. Accordingly, constructing integrated solid‐solid interface is as important as promoting the ionic conductivity to present a well performed SSLB. To this end, in situ polymerization can be a promising strategy which has been verified in our previous work.^[^ ^15^ ^]^ In this procedure, the liquid monomer precursors which possess the ability to permeate and thus create fine interfacial contact at essentially all length scales are firstly injected into a cell. Subsequent polymerization transforms the liquid precursors to a SPE, retaining the fine interfacial characteristics and thereby integrating the solid–solid interface, which favorably address the notorious solid‐state contact issue.^[^ ^16^ ^]^

**Figure 1:**
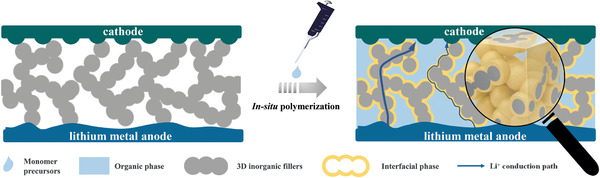
Schematic illustration of the in situ integrated SSLB with 3D framework.

Inspired by the above scenarios, here, a novel integrating strategy is presented to demonstrate a successful SSLB: the SPE monomer precursors are casted into self‐supported porous SIE framework, namely, the three‐dimensional (3D) fillers, then in situ polymerized to develop rigid–flexible coupling composite and meanwhile to integrate electrolyte/electrode interface.^[^ ^17^, ^18^ ^]^ As illustrated in **Figure** **1**, in such configuration, the 3D framework can build fast Li^+^ percolated pathway along both the SIE phase and increase the SIE/SPE interfacial area to improve the interface conductivity,^[^ ^19^ ^]^ while the in situ polymerization integrates the electrolyte/electrode interface successfully. To carry out this strategy, highly conductive LATP is used to prepare self‐supported porous framework (p‐LATP). Poly(ethylene glycol) methyl ether acrylate (PEGMEA, 
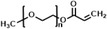
 *n* = 8−9) is used as the precursors for in situ polymerization [poly(PEGMEA), P(PEGMEA)] due to its good compatibility with lithium metal.^[^ ^20^, ^21^ ^]^ It is demonstrated that p‐LATP/P(PEGMEA) coupling composite (the 3D composite) possesses a conductivity of 2.0 × 10^−4^ S cm^−1^ at room temperature, which is comparable to that of the dense LATP. The in situ polymerized P(PEGMEA) can not only integrate the loose interfacial contact but also protect LATP from being reduced by lithium metal, which is of significance for high energy lithium batteries. As a consequence, the interfacial resistance decreases significantly from 6502 to 167 Ω cm^2^. The LiCoO_2_ cathode based SSLB employing lithium metal anode delivers a superior capacity of 143 mA h g^−1^ (3.0−4.3 V vs Li^+^/Li) at 0.1 C and outstanding capacity retention of 82% after 120 cycles with an average Coulombic efficiency of 98.4%. Moreover, the LiNi_0.8_Mn_0.1_Co_0.1_O_2_ (NMC811)|Li SSLB based on 3D composite presents a satisfactory capacity of 172 mA h g^−1^ (3.0−4.5 V vs Li^+^/Li) at 0.2 C and good capacity retention of 75% after 50 cycles at room temperature. The innovative work of *in‐situ* integrated porous SIE is hence a big leap for the development of SSLB, which is expected to address the bottlenecks of poor ionic conductivity at ambient temperature and huge interfacial resistance.

## 2 Results and Discussion

### 2.1 Characterization of p‐LATP and the 3D Composite

**Figure 2:**
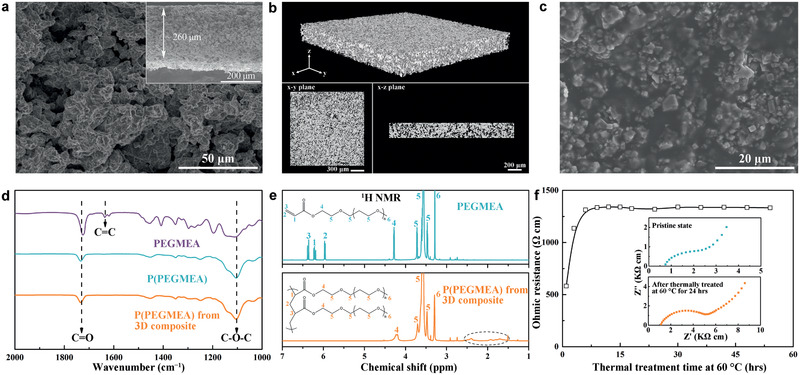
Characterization of p‐LATP and the 3D composite. a) SEM cross‐sectional view of p‐LATP. Inset shows the cross‐sectional view of p‐LATP at low magnification. b) The 3D reconstruction image of p‐LATP and corresponding 2D sliced images from x−y, x−z plane. The gray and black represent LATP phase and pores, respectively. c) SEM cross‐sectional view of the 3D composite. d) FTIR spectra of PEGMEA and P(PEGMEA) with or without the 3D composite. e) ^1^H NMR spectra of PEGMEA and P(PEGMEA) from the 3D composite. The solvents are deuterated *N*,*N*‐dimethylformamide. Insets show the corresponding structural formula of PEGMEA and P(PEGMEA). f) Thermal evolution of ohmic resistance at 60 °C for steel|3D composite|steel symmetrical cell. Inset shows the room‐temperature EIS plots of the steel|3D composite|steel symmetrical cells before and after being thermally treated at 60 °C for 24 h.

The synthesized LATP powders are well crystallized verified by X‐ray diffraction (XRD) results shown in Figure S1a (Supporting Information), which also demonstrates that the XRD pattern of p‐LATP is well consistent with that of the as prepared powders, implying all the graphite has been removed during high temperature sintering. A self‐supported highly porous p‐LATP with thickness of about 260 µm is demonstrated clearly by scanning electron microscopy (SEM) imaging in **Figure** **2**a. The microstructure characteristics of p‐LATP are further characterized via a mercury injection method. As shown in Figure S1b (Supporting Information), the pore diameter demonstrates bimodal distribution in the range of 1−10 µm with average value of 7.40 and 3.67 µm. The porosity of p‐LATP is tested to be 54% with pore tortuosity of 1.62. To further reveal the porous microstructure of p‐LATP, the X‐ray computed tomography is conducted. The uniform pore distribution observed in both the 3D reconstruction image of p‐LATP and corresponding two dimensional (2D) sliced images from x−y, x−z plane proves p‐LATP with percolated porous structure has been fabricated successfully. According to the electrochemical impedance spectroscopy (EIS) results in Figure S1c (Supporting Information), the ionic conductivity of p‐LATP at room temperature is calculated to be 2.6 × 10^−5^ S cm^−1^. Though this value is one order of magnitude lower than that of the dense LATP (3.4 × 10^−4^ S cm^−1^) due to its high porosity, it is still one order of magnitude higher than that of PEO. The large pore size, high porosity, and small pore tortuosity render p‐LATP satisfactory microstructure characteristics for monomer penetrating and thus in situ integrating the 3D composite inside SSLB feasibly.

After thermally treating the PEGMEA and p‐LATP mixture at 60 °C for 24 h, Figure S2a (Supporting Information) displays the solidification of PEGMEA, indicating the successful polymerization of P(PEGMEA) within the 3D composite. To vividly observe the inner state of the 3D composite, cross‐sectional SEM imaging is conducted. As shown in Figure 2c, the as prepared 3D composite presents continuous LATP surrounded by uniformly distributed P(PEGMEA) with no presence of pores. The uniform distribution of Ti, P, Al, C, and O elements also ensures the homogeneous 3D composite without LATP aggregation, indicating the positive role of p‐LATP (Figure 2c and Figure S2b, Supporting Information).

Fourier transform infrared spectrometer (FTIR) and nuclear magnetic resonance (NMR) are used to check the molecular structure of the 3D composite. After being thermally treated at 60 °C for 24 h, Figure 2d shows that the C=C functional group at around 1630 cm^−1^ disappears while other functional groups, e.g., C=O or C—O—C, remain unchanged, implying the successful polymerization of PEGMEA into P(PEGMEA). The FTIR spectrum of P(PEGMEA) from the 3D composite is the same as that of the pristine P(PEGMEA), which means that LATP has no influence on PEGMEA polymerization and no side reactions happen between LATP and PEGMEA. Figure 2e indicates that the signals located at 6.4, 6.2, and 6.0 ppm are ascribed to the hydrogen atoms connected to C=C bond in PEGMEA. After thermally treated, these peaks disappear while several looming wrinkles in the range 2.5−1.5 ppm emerge, suggesting the polymerization of PEGMEA, which can also be verified by the ^13^C spectra in Figure S3 (Supporting Information). Both FTIR and NMR results indicate that the polymerization of PEGMEA can be successfully carried out within p‐LATP.

**Figure 3:**
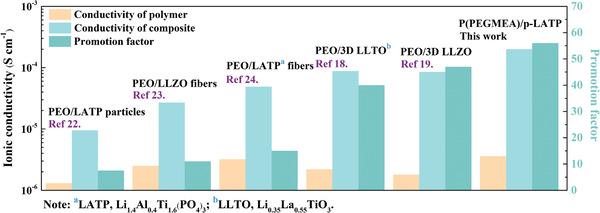
Ionic conductivity and the promotion factor comparison of different solid electrolytes.^[^ ^18^, ^19^, ^22^, ^23^, ^24^ ^]^ The promotion factor is defined as the ratio of the composite conductivity to the conductivity of polymer.

The resistance change with thermal treatment time can also be an effective criterion of polymerization. Figure 2f plots the relationship between the ohmic resistance of a steel|3D composite|steel symmetrical cell and thermal treatment time at 60 °C. The resistance increases substantially from 582 to 1335 Ω cm (60 °C) in the first 9 h due to the polymerization of PEGMEA to increase the energy barrier of Li^+^ transportation in polymer matrix. After that, the resistance remains unchanged owing to the successful polymerization of PEGMEA. According to the ohmic resistance and EIS plots (Figure S4a, Supporting Information), the ionic conductivity of the 3D composite is calculated to be 2.0 × 10^−4^ S cm^−1^ at room temperature. This value is comparable to that of the dense LATP, 3.4 × 10^−4^ S cm^−1^, and 23 times higher than that of P(PEGMEA)/zero dimensional (0D) LATP composite, 8.6 × 10^−6^ S cm^−1^ (Figure S4b, Supporting Information), suggesting fast and efficient ion transport of the porous framework. As shown in **Figure** **3**, when using 0D powders or one dimensional (1D) fibers as fillers,^[^ ^22^, ^23^, ^24^ ^]^ ionic conductivity of the polymer can be promoted by a factor of 7.5−15. By replacing the 0D powders or 1D fibers with 3D LLTO,^[^ ^18^ ^]^ the promotion factor increases significantly to 40 corresponding to the increase of ionic conductivity from 2.2 × 10^−6^ to 8.8 × 10^−5^ S cm^−1^. With the contribution of highly conductive p‐LATP designed by this work, the promotion factor can be increased further to 56 with a high ionic conductivity of 2.0 × 10^−4^ S cm^−1^, implying the great impact of continuous Li^+^ transportation pathway stemmed from p‐LATP and the newly generated LATP/P(PEGMEA) interface.

### 2.2 Origin of the Great Conductivity Enhancement

**Figure 4:**
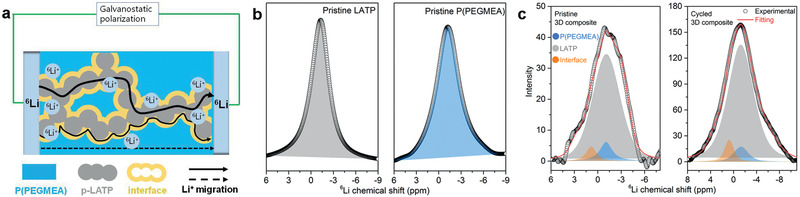
Li^+^ migration behavior within the 3D composite. a) Schematic representation of Li^+^ migration in the 3D composite. b) ^6^Li SSNMR spectra of the pristine LATP and P(PEGMEA). c) ^6^Li SSNMR spectra of the pristine and as‐cycled 3D composite.

Solid‐state NMR (SSNMR) is one vogue method to illuminate the Li^+^ migration behavior within composite solid electrolytes. To explain why the ionic conductivity of P(PEGMEA) is enhanced significantly after compositing with p‐LATP, SSNMR is conducted coupled with a ^6^Li/^7^Li isotope‐replacement method with ^6^Li metal as lithium source (**Figure** **4**a). During unidirectional galvanostatic polarization, ^6^Li^+^ is stripped from one ^6^Li metal and plated on the other, passing through the 3D composite. It can be concluded that the more favorable pathway the Li^+^ migrates along, the more significant evolution of ^6^Li (or ^7^Li) will be. Consequently, the preferential Li^+^ migration pathway can be speculated via comparing ^6^Li or ^7^Li evolution of different local environment before and after polarization.

SSNMR is firstly conducted on pristine P(PEGMEA) and LATP (Figure 4b) to distinguish the ^6^Li located in variable local environments. The ^6^Li resonance in LATP is at about −1.27 ppm while this value for pristine P(PEGMEA) is about −1.23 ppm. Based on the lithium contents, i.e., 46 vol% LATP versus 54 vol% P(PEGMEA) with 0.9 m Li^+^, the ^6^Li content in LATP and P(PEGMEA) phase is firstly quantified to be 198.0 and 24.6, respectively (Figure 4c). According to previous reports,^[^ ^17^, ^18^, ^19^, ^25^, ^26^ ^]^ the new peak at 0.87 ppm can be attributed to the Li^+^ located in LATP/P(PEGMEA) interfacial phase, which can also be verified by the differential scanning calorimetry results. As shown in Figure S5a (Supporting Information), P(PEGMEA) shows the glass transition temperature (*T*_g_) at −62 °C, which is consistent with previous report.^[^ ^21^ ^]^ After compositing with 0D LATP powders, the *T*_g_ of P(PEGMEA) increases to −54 °C, suggesting the generation of LATP/P(PEGMEA) interfacial phase.^[^ ^27^ ^]^ With p‐LATP, no filler aggregation happens and thus larger interfacial area is formed, leading to an advanced increase of *T*_g_ to −25 °C. After polarized for 2 h at a current density of 60 µA cm^−2^ (Figure S5b, Supporting Information), the ^6^Li content (peak area) of LATP, P(PEGMEA), and interface increases to 1100.4, 69.2, and 62.9 with the growth of 456, 181, and 314%, respectively. The great enhancement of ^6^Li content verifies the highly conductive essence of LATP/P(PEGMEA) interface. Combined with the continuous highly conductive p‐LATP, the ionic conductivity of the 3D composite can hence increase significantly to be comparable with LATP.^[^ ^1^, ^2^ ^]^

### 2.3 Compatibility with Lithium Metal

The battery's performance depends strongly on the compatibility between electrolyte and electrode.^[^ ^28^ ^]^ To this end, the compatibility of the 3D composite with lithium metal is demonstrated in Li−Li symmetrical cells with alone P(PEGMEA) and LATP electrolytes for fair comparison. Typical EIS plots and equivalent circuit models of pristine and after thermally treated Li−Li symmetrical cells based on different electrolytes are presented in Figure S6 (Supporting Information). While, the refined parameters are shown in Table S1 (Supporting Information). EIS plots of the pristine Li|PEGMEA|Li and Li|LATP|Li symmetrical cells are comprised of one semicircle with a tail connected. It has been previously reported, that in this case,^[^ ^29^, ^30^ ^]^ the ohmic resistance is determined by the high frequency intercept with the real axis while the interfacial resistance is represented by the span of the semicircle. For the battery with the 3D composite, another semicircle emerges at high frequency, which probably comes from the contribution of the solid‐state p‐LATP. After thermally treated at 60 °C for 24 h, the EIS plot of Li|P(PEGMEA)|Li cell also shows a high‐frequency semicircle due to the polymerization of PEGMEA. It can be concluded that the semicircle at high frequency is the response to the ohmic resistance and the semicircle at middle frequency represents the interfacial resistance.^[^ ^31^ ^]^

**Figure 5:**
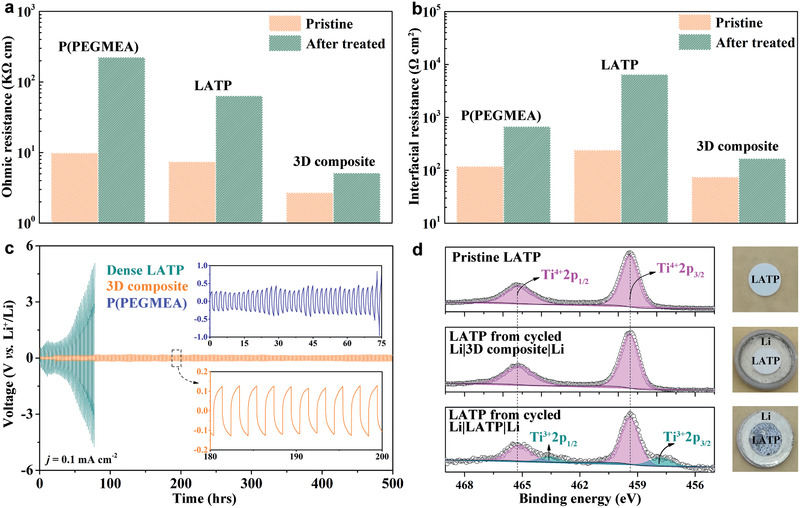
Compatibility of the 3D composite with lithium metal. a) Ohmic and b) interfacial resistance calculated from the EIS plots. c) D.C. galvanostatic cycle of Li−Li symmetrical cells based on P(PEGMEA), LATP, and the 3D composite at room temperature. The area specific capacity is 0.1 mA h cm^−2^ at a current density of 0.1 mA cm^−2^. d) Ti 2p XPS and digital images of the pristine LATP and cycled LATP in the Li|3D composite|Li and Li|LATP|Li symmetric batteries.

According to the fitting results, the ohmic and interfacial resistance of the Li−Li symmetrical cells are shown in **Figure** **5**a,b, respectively. The ohmic resistance of the pristine Li|3D composite|Li symmetrical cell is 2710 Ω cm, significantly smaller than that of the pristine Li|PEGMEA|Li cell, 9987 Ω cm. After being thermally treated, the ohmic resistance of Li|3D composite|Li cells increases to 5142 Ω cm while the value of Li|P(PEGMEA)|Li increases sharply to 223 969 Ω cm. Meanwhile, the interfacial resistance of Li|3D composite|Li cell increases from 75 to 167 Ω cm^2^, 4 times lower than that of the Li|P(PEGMEA)|Li cell, 673 Ω cm^2^. The ohmic and interfacial resistance comparison between the 3D composite and P(PEGMEA) electrolytes emphasizes the significance of continuous Li^+^ conducting pathway stemmed from p‐LATP, which can be protected by P(PEGMEA) from being reduced by lithium metal as verified by the resistance behavior in Li|LATP|Li battery shown in Figure S6b,e (Supporting Information), that the ohmic resistance increases from 7426 to 63 802 Ω cm and the interfacial resistance increases from 243 to 6502 Ω cm^2^ after thermally treated, in good accordance with the direct current (D.C.) polarization results shown in Figure 5c discussed below.

The compatibility of P(PEGMEA), LATP, and the 3D composite with lithium metal is further examined via D.C. polarization at room temperature. In Figure 5c, a flat voltage plateau of around 0.13 V for over 500 h is observed at a current density of 0.1 mA cm^−2^ for Li|3D composite|Li. Moreover, the interfacial resistance of Li|3D composite|Li slightly increases after cycling in Figure S7b (Supporting Information), implying the good compatibility of 3D composite with lithium metal. For Li|P(PEGMEA)|Li, the large voltage of 0.27 V at the beginning and 0.81 V after 75 h in can be attributed to the large polarization resistance due to the poor ionic conductivity (3.6 × 10^−6^ S cm^−1^) and the low Li^+^ transference number (0.12, Figure S9, Supporting Information) of P(PEGMEA). By a sharp contrast, the voltage of Li|LATP|Li increases continuously to 5 V in only 77 h, agreeing well with the resistance behavior in Figure S7 (Supporting Information). To clarify the failure mechanism of Li|LATP|Li, the as‐cycled battery is disassembled to check the inner state. Figure 5d displays that the LATP from the as‐cycled Li|LATP|Li battery are blackened while the p‐LATP from the Li|3D composite|Li battery still presents the natural color (white) of LATP, suggesting the potential reactions when LATP is in contact with Li. To validate this, X‐ray photoelectron spectroscopy (XPS) analysis is conducted on the blackened LATP and p‐LATP from the 3D composite with pristine LATP for fair comparison. For the pristine LATP, the peaks at 465.3 and 459.4 eV represent Ti^4+^. After cycling in Li|LATP|Li, namely, for the blackened LATP, new peaks of 463.6 and 457.8 eV belong to Ti^3+^ have been detected. According to the area simulation, the ratio of Ti^4+^ to Ti^3+^ is estimated to be around 4:1. No valance variation is found for Al^3+^ or P^5+^ (Figure S10, Supporting Information). Based on the first‐principles calculation and XPS results, a possible chemical reaction between LATP and Li is

(1) $$\begin{array}{ccc} & & 10Li_{1.3}Al_{0.3}Ti_{1.7}{\left(PO_{4}\right)}_{3}\;+17\mathrm{Li}\to 3\mathrm{AlP}O_{4}\\ & & \;+\;10Li_{3}PO_{4}+17\mathrm{TiP}O_{4},\;\Delta G=-0.321\;\;\mathrm{eV} \end{array}$$

The negative Gibbs free energy indicates that LATP can react with Li spontaneously to produce products with higher resistance (than LATP) and thereby leading to the failure of Li|LATP|Li. By integrating p‐LATP with in situ polymerized P(PEGMEA), no Ti^3+^ signals are detected from the XPS results, which implies that these negative reactions are avoided due to the P(PEGMEA) protective layer between p‐LATP and Li, ensuring a stable operating Li|3D composite|Li battery. Thus, the 3D composite is compatible with lithium metal while the alone P(PEGMEA) or LATP are not.

### 2.4 Demonstration in SSLB

**Figure 6:**
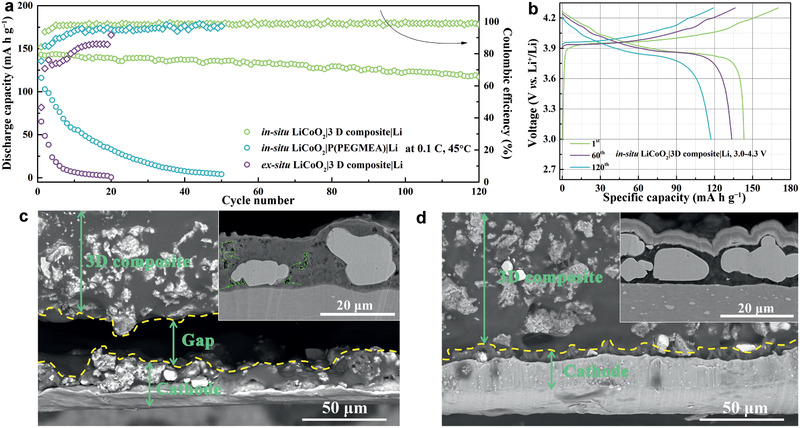
a) Cycle performance of in situ LiCoO_2_|3D composite|Li, in situ LiCoO_2_|P(PEGMEA)|Li, ex situ LiCoO_2_|3D composite|Li SSLBs working at 3.0−4.3 V versus Li^+^/Li. b) Charge–discharge curves of in situ LiCoO_2_|3D composite|Li SSLB at the rate of 0.1 C. Typical SEM cross‐sectional view of the LiCoO_2_/3D composite interface from the disassembled c) ex situ and d) in situ LiCoO_2_|3D composite|Li SSLBs. Insets show the SEM cross‐sectional view of the cathodes.

The feasibility of the 3D composite is examined in SSLBs using LiCoO_2_ or NMC811 cathodes and lithium metal anode. To verify the role of integrated electrolyte/electrode interface, the performance of SSLBs fabricated via ex situ processes is conducted for a fair comparison. For the in situ integrated LiCoO_2_|3D composite|Li SSLB operated at 3.0−4.3 V versus Li^+^/Li, an initial discharge specific capacity of 143 mA h g^−1^ is presented at the rate of 0.1 C, corresponding to an initial Coulombic efficiency of 84% (**Figure** **6**a,b). With increasing rate to 0.2, 0.5, and 1 C, the discharge capacity decreases to 126, 104, and 78 mA h g^−1^, respectively (Figure S11, Supporting Information). With an average Coulombic efficiency of over 98.4%, this *in‐situ* integrated SSLB with the 3D composite can cycle stably for 120 cycles with high capacity retention of 82% at 0.1 C. In contrast, the initial discharge capacity of the in situ LiCoO_2_|Li battery based on P(PEGMEA) is 116 mA h g^−1^, which undergoes rapid decay to zero within 50 cycles. Even worse, the LiCoO_2_|3D composite|Li SSLB fabricated by the ex situ method survives no more than 20 cycles with the initial discharge capacity of only 65 mA h g^−1^ at 45 °C.

It can be speculated that the inferior performance of the in situ integrated LiCoO_2_|P(PEGMEA)|Li SSLB mainly originates from the low ionic conductivity of P(PEGMEA), i.e., 1.0 × 10^−5^ S cm^−1^ at 45 °C (Figure S4, Supporting Information), to result in sluggish kinetics. To explain the reason for the failure of the ex situ LiCoO_2_|P(PEGMEA)|Li SSLB, SEM imaging are conducted to observe the microstructure difference between the ex situ and in situ SSLB. For the ex situ SSLB, a gap of about 30 µm is seen clearly between the 3D composite and cathode (Figure 6c). In depth, many voids are shown within the cathode (marked by green dashed line). Such poor contact blocks the Li^+^ transportation severely, leading to large battery resistance and poor electrochemical performance with cycling (Figure S13a, Supporting Information and Figure 6a). While, the SSLB fabricated via the *in‐situ* processes can form integrated structure not only at the electrolyte/cathode interface but also within the cathode (Figure 6d) and thereby guaranteeing fast Li^+^ migration to endow moderate battery resistance (Figure S13b, Supporting Information). Based on the performance comparison among the three kinds of SSLBs, it can be concluded that both the high ionic conductivity and integrated electrolyte/electrode interface are inevitable factors to a well performed SSLB. Encouraging, these key factors can be realized by in situ integrating SSLBs using 3D composite presented by this work.

**Figure 7:**
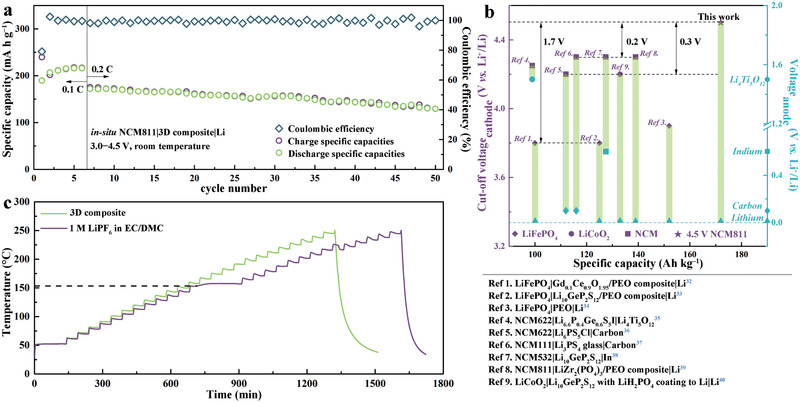
a) Cycle performance of in situ NMC811|3D composite|Li SSLB working at 3.0−4.5 V versus Li^+^/Li at room temperature. b) The electrochemical performance of SSLBs using different solid electrolytes and electrodes.^[^ ^32^, ^33^, ^34^, ^35^, ^36^, ^37^, ^38^, ^39^, ^40^ ^]^ c) Temperature−time curves of accelerating rate calorimetry heat‐wait‐search mode for fully charged NMC811|Li batteries based on the 3D composite and 1 m LiPF_6_ in EC/DMC (v:v = 1:1).

With the 3D composite, even at a higher working voltage of 3.0−4.5 V (vs Li^+^/Li), the in situ NMC811|3D composite|Li SSLB can deliver a satisfactory capacity of 172 mA h g^−1^ at 0.2 C and good capacity retention of 75% after 50 cycles with an average Coulombic efficiency of 99.1% at room temperature (**Figure** **7**a). Figure 7b compares the electrochemical performance of the previously reported SSLBs based on various electrolytes and electrodes.^[^ ^32^, ^33^, ^34^, ^35^, ^36^, ^37^, ^38^, ^39^, ^40^ ^]^ With the lithium metal as anode, the in situ NMC811|3D composite|Li SSLB has the highest cut‐off voltage than that of other SSLBs. For example, it is 1.7 V higher than LiFePO_4_, 0.25 V higher than LiCoO_2_, and 0.2 V higher than NMC, corresponding increase of discharge capacities of 34, 42, and 14 mA h g^−1^, respectively. It is commonly acknowledged that a high cut‐off voltage means a high energy density. Accordingly, the integrated strategy proposed herein is very promising to design SSLB with high energy density.

Although the in situ NMC811|3D composite|Li SSLB possesses high energy density, it still demonstrates superior safety characteristic. To verify this, the accelerating rate calorimetry with heat‐wait‐search mode is used to reveal the superior thermal stability of a fully charged NMC811|3D composite|Li SSLB with a liquid battery based on 1 M LiPF_6_ in ethylene carbonate (EC)/dimethyl carbonate (DMC) for fair comparison. Figure 7c shows the self‐heating onset temperature of the liquid battery is about 153°C, suggesting the decomposition of 1 m LiPF_6_‐EC/DMC at the relatively low temperature. For SSLB, the self‐heating is not detected even at 250°C, which is one hundred degrees Celsius higher than that of the liquid battery and hence indicating the superior safety feature of the high energy density SSLB endowed by this novel integrated strategy.

## 3 Conclusion

In summary, a highly conductive 3D composite is carefully designed by in situ polymerizing PEGMEA precursors within p‐LATP. The continuous LATP and generated interfacial phase can provide fast Li^+^ migration pathway, endowing the 3D composite fantastic conductivity of 2.0 × 10^−4^ S cm^−1^ at room temperature. While the in situ polymerized PEGMEA can integrate the loose interface contact and protect the LATP from being reduced by lithium metal anode, decreasing the interfacial resistance of SSLB from 6502 to 167 Ω cm^2^. The SSLBs utilizing high voltage LiCoO_2_, NMC811 cathodes and lithium metal anode exhibit high initial discharge capacity of 143 and 190 mA h g^−1^ and satisfactory capacity retention of 82% after 120 cycles and 75% after 50 cycles, respectively. The presentation suggests that this novel strategy can provide significant guidance on designing highly conductive solid electrolyte that demonstrates superior electro‐chemical compatibility with high voltage cathode and lithium metal anode to significantly boost the development of practical high energy SSLBs.

## 4 Experimental Section

LATP powders were synthesized by a solid‐state reaction method. In detail, stoichiometric amounts of LiOH (MACKLIN, 99.9%), Al_2_O_3_ (Sinopharm Chemical Reagent Co., Ltd, AR), TiO_2_ (aladdin, 99.8%), and NH_4_H_2_PO_4_ (Sinopharm Chemical Reagent Co., Ltd, GR) were mixed according to the formula by planetary ball‐milling using isopropanol (Sinopharm Chemical Reagent Co., Ltd, AR) as medium at a rotation speed of 350 rpm min^−1^ for 12 h. Then, the slurry was dried at 60 °C for 12 h and sintered at 450 °C for 20 h. The obtained bulk materials were grounded into powders and sintered at 850 °C for another 10 h to obtain LATP powders. To prepare p‐LATP, LATP powders were mixed with graphite powders (MACKLIN, 99.95%) with a weight ratio of 1:1. Then, the mixtures were pressed into green pellets under a pressure of 12 MPa and sintered at 850 °C for 10 h at a heating rate of 3 °C min^−1^ in air. The porosity and pore size distribution of p‐LATP were measured using a mercury injection apparatus (Quantachrome PM60GT‐17). To fabricate dense LATP pellets, LATP powders were pressed into green pellets under a pressure of 12 MPa and sintered at 850 °C for 10 h at a heating rate of 3 °C min^−1^ in air. PEGMEA precursor solution was prepared via dissolving 0.13 g lithium difluoro(oxalato)borate in 1 g PEGMEA, resulting in a Li^+^ concentration of 0.9 m. Then, 5 mg azodiisobutyronitrile is added into the solution to initiate PEGMEA polymerization.

To verify the positive role of in situ integration processes, that is, the role of an integrated electrolyte/electrode interface, the performance of SSLBs fabricated via both in situ and ex situ processes were compared. To fabricate in situ cathode (LiCoO_2_ or NMC811)|Li SSLBs based on the 3D composite or P(PEGMEA) electrolytes, p‐LATP or cellulose separator were firstly put on one lithium metal disc, then 100 µL of PEGMEA solution is added into p‐LATP or cellulose separator. The cathode@Al foil was then put on the top of p‐LATP or cellulose separator to finish the assembly of CR2032 cells. At last, these cells were transferred into a 60 °C oven to initiate the polymerization of PEGMEA. To fabricate ex situ LiCoO_2_|3D composite|Li SSLB, p‐LATP is firstly put on a poly(tetrafluoroethylene) plate, then 100 µL PEGMEA solution was added into p‐LATP. After polymerization, the 3D composite was moved from the plate and sandwiched between lithium metal disc and LiCoO_2_@Al foil to finish the assembly of CR2032 cells. All the fabrication processes were conducted in an argon‐filled glovebox.

Other experimental details can be found in the Supporting Information.

## Conflict of Interest

The authors declare no conflict of interest.

## Supporting information

Suppporting Information

## Data Availability

The data that support the findings of this study are available from the corresponding author upon reasonable request.
