# Supplementary material for: In Situ Polymerization Permeated Three‐Dimensional Li+‐Percolated Porous Oxide Ceramic Framework Boosting All Solid‐State Lithium Metal Battery
Source: Adv Sci (Weinh). 2021 Mar 3;8(9):2003887. doi: 10.1002/advs.202003887 (PMC8097327; doi:10.1002/advs.202003887)
Supplement: Supplementary file 1 — Suppporting Information [file ADVS-8-2003887-s001.pdf]

WILEY-VCH

© 2021 Wiley-VCH GmbH

ADVANCED SCIENCE
Open Access

Supporting Information

for *Adv. Sci.*, DOI: 10.1002/advs.202003887

In Situ Polymerization Permeated Three-dimensional Li+-percolated Porous oxide Ceramic Framework Boosting all Solid-state Lithium Metal battery

*Yiyuan Yan‡, Jiangwei Ju‡, Shanmu Dong, Yantao Wang, Lang Huang, Longfei Cui, Feng Jiang, Yanfen Zhang, and Guanglei Cui**

Supporting Information

***In situ* polymerization permeated three-dimensional Li^+-percolated porous oxide ceramic framework boosting all solid-state lithium metal battery**

*Yiyuan Yan‡, Jiangwei Ju‡, Shanmu Dong, Yantao Wang, Lang Huang, Longfei Cui, Feng Jiang, Yanfen Zhang, and Guanglei Cui**

Y. Yan, J. Ju, S. Dong, Y. Wang, L. Huang, L. Cui, F. Jiang, Q. Wang, Y. Zhang, Prof. G. Cui
Qingdao Industrial Energy Storage Research Institute, Qingdao Institute of Bioenergy and Bioprocess Technology, Chinese Academy of Sciences, Qingdao 266101, People's Republic of China
‡Contributed equally
*Corresponding author: cuigl@qibebt.ac.cn

Experiments

Preparation of cathodes

The $LiCoO_2$ cathodes were fabricated as follows: $LiCoO_2$, super P carbon, and polyvinylidene fluoride (PVDF) were mixed according to a weight of 8:1:1 in NMP solvent. Then, the slurry was casted on Al foil. Subsequently, the cathodes were moved into a 60 °C oven for 24 hours to remove the solvent. At last, the cathode@Al foil was punched into disk in diameters of 10 mm. Loading of the active material was 1.46 ± 0.01 mg cm^{-2}.

The $LiNi_{0.8}Mn_{0.1}Co_{0.1}O_2$ (NMC811) cathodes were fabricated as follows: NMC811, super P carbon, and polyvinylidene fluoride (PVDF) were mixed according to a weight of 8:1:1 in NMP solvent. Then, the slurry was casted on Al foil. Subsequently, the cathodes were moved into a 60 °C oven for 24 hrs to remove the solvent. At last, the cathode@Al foil was punched into disk in diameters of 10 mm. Loading of the active material was 1.89 ± 0.01 mg cm^{-2}.

Battery assembly

To fabricate steel|steel symmetrical cell, the porous LATP substrate or cellulose separator were firstly put on one steel disc, then 100 μL of PEGMEA solution was added into the substrate or cellulose separator. At last, another steel disc was put on the top of the substrate or cellulose separator to finish the assembly of CR2032 cells. The Li|Li symmetrical cells were prepared via a similar procedure by replacing steel disc with Li metal disc. To fabricate the cell to the test cyclic voltammetry (CV), the porous LATP substrate or cellulose separator were firstly put on one Li metal disc, then 100 μL of PEGMEA solution was added into the substrate or cellulose separator. At last, a steel disc was put on the top of the substrate or cellulose separator to finish the assembly of CR2032 cells.

The steel|P(PEGMEA)/0D LATP composite|steel symmetrical cell was assembled to compare with 3D composite. The PEGMEA-LATP slurry was obtained via dispersing 70 wt% LATP powder into the PEGMEA solution. The P(PEGMEA)/0D LATP composite obtained via tape casting process by doctor blading the slurry on the poly(tetrafluoroethylene) plate. After polymerization, the electrolyte was peeled off with blade and sandwiched between steel discs.

All these processes were conducted in an Ar-filled glove box. After assembly, all the cells containing PEGMEA were transferred into a 60 °C oven to initiate the polymerization of PEGMEA.

Characterization

LATP crystal phase was identified by X-ray diffraction (XRD, Bruker D8 ADVANCE) analysis. Scanning electron microscopy (SEM, Hitachi S-4800) was used to observe the microstructure morphologies of different kinds of electrolytes. The internal structure of the

p-LATP is characterized by X-ray computed tomography (CT, Rigaku CT lab HX130). The nuclear magnetic resonance (NMR, Bruker AVANCE III 600 MHz) and Fourier transform infrared spectrometer (FTIR, Bruker VERTEX 70) were used to analyze the molecular structures of PEGMEA, P(PEGMEA), and 3D composite.

Differential scanning calorimetry (DSC) measurements were carried out with a heating rate of 5 °C min^{-1} from −100 to 10 °C under N_2 atmosphere. ^6Li magic angle spinning solid-state NMR is performed on a 20.0 T (850 MHz) Varian VNMRS spectrometers with a ^6Li Larmor frequency of 125 MHz. P(PEGMEA), LATP, and the 3D composite are packed in 4.0 mm rotors and spun at a speed of 8 kHz. For ^6Li NMR, the 90° pulse length is 5 μs. 64 scans are acquired for the LATP, P(PEGMEA) and 3D composite with a recycle delay of 600 s. Chemical shifts are referenced to 1 M LiCl solution at 0 ppm.

The analysis of major element of LATP and 3D composite before and after cycling in Li|Li symmetric cells was conducted with X-ray photoelectron spectroscopy (XPS, Thermo Scientific ESCA Lab 250Xi). The thermal stability of 3D composite and conventional 1 M $LiPF_6$ in EC/DMC (v: v=1:1) electrolyte was tested by ARC (BTC130, HEL Company, Hertfordshire, UK).

ARC measurements were conducted by the mode of HWS with detection limit (for exotherm) of 0.03 °C min^{-1}. The starting temperature was 50 °C with a heating step of 10 °C. The detected self-heating rate was 0.03 °C min^{-1} and the waiting time was 40 min.

The ionic conductivity of dense LATP pellet was estimated using electrochemical impedance spectroscopy (EIS) method. To do this, colloidal graphite was firstly smeared on two sides of the LATP pellet as current collector. The conductivity of P(PEGMEA),

P(PEGMEA)/0D LATP composite and 3D composite was measured in steel|steel symmetrical CR2032 cells. EIS measurements were conducted in the frequency range of 7 MHz to 1 Hz at room temperature depending on different conditions. Lithium ion transference number (t_{Li^+}) of the 3D composite and P(PEGMEA) was measured in Li|Li symmetrical CR2032 cells according to,

$$t_{Li^+} = I^{ss}R_{ohm}^{ss}(\Delta V - I^o R_p^o)/I^o R_{ohm}^o(\Delta V - I^{ss}R_p^{ss}) \quad (1)$$

where ΔV is the applied amplitude voltage, 50 mV, and the superscripts of o and ss represent the initial and steady state, respectively. I is the current; R_{ohm}, and R_p are the ohmic and interfacial resistance of the symmetrical cells, respectively. The CV test was conducted at a scanning rate of 1 mV s^{-1}. The DC polarization tests of different electrolytes were conducted at a current density of 0.1 mA cm^{-2}. The cycle performance of lithium battery with $LiCoO_2$ cathodes was acquired at 0.1 C with voltage range from 3.0 to 4.3 V. The rate performance of lithium battery with $LiCoO_2$ cathodes was acquired at 0.05 C, 0.1 C, 0.2 C, 0.5 C and 1 C with voltage range from 3.0 to 4.3 V (1 C=140 mA h g^{-1}). The cycle performance of lithium battery with NMC811 cathodes was acquired at 0.2 C with voltage range from 3.0 to 4.5 V (1 C=180 mA h g^{-1}).

Galvanostatic charge−discharge tests and DC polarization tests were carried out via LAND testing system (Wuhan LAND Electronics Co., Ltd.).

Table S1. Fitting results of EIS analysis for pristine Li|PEGMEA|Li, Li|LATP|Li, Li|3D composite|Li and after treated Li|P(PEGMEA)|Li, Li|LATP|Li, Li|3D composite|Li symmetric cells.

		R_{ohm} (Ω cm)	R_p (Ω cm^2)	C_p (F)
pristine	Li|PEGMEA|Li	9887	119	1.5×10^{-5}
	Li|LATP|Li	7426	243	4.6×10^{-7}
	Li|3D composite|Li	2710	75	4.9×10^{-6}
after treated	Li|P(PEGMEA)|Li	223969	673	6.4×10^{-6}
	Li|LATP|Li	63802	6502	7.8×10^{-8}
	Li|3D composite|Li	5142	167	3.4×10^{-6}

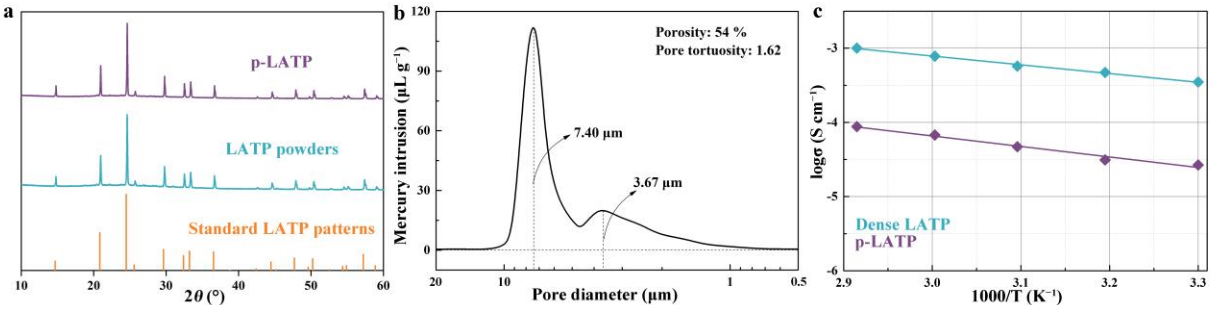

Figure S1. **(a)** XRD patterns of standard LATP, the as-prepared LATP powders, and p-LATP. **(b)** The pore size distribution of p-LATP. **(c)** Relation between ionic conductivity of electrolyte and temperature for dense LATP and p-LATP.

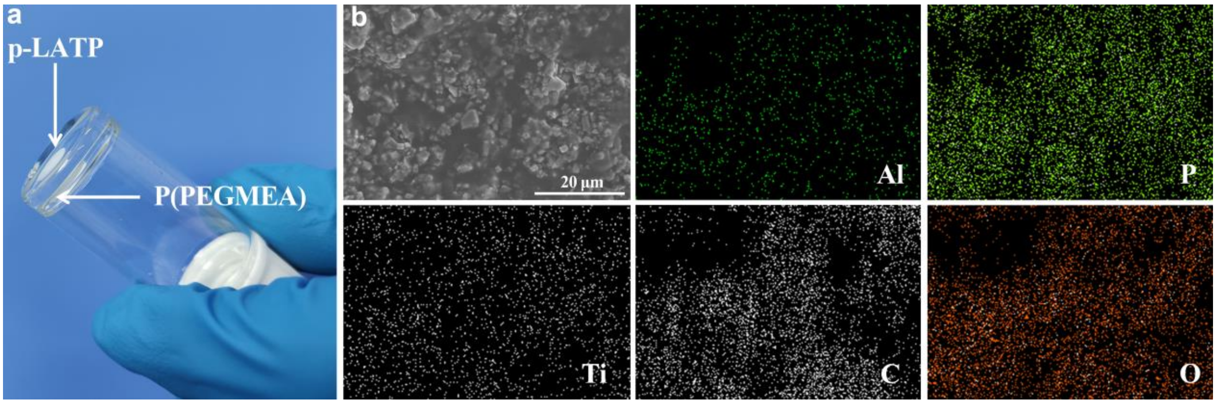

Figure S2. (**a**) Digital image of PEGMEA with p-LATP after being thermally treated at 60 °C for 24 hrs. (**b**) SEM cross-sectional view and element mapping analysis of the 3D composite.

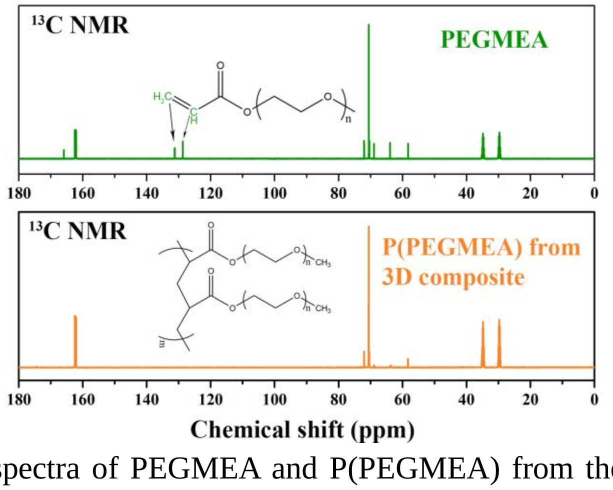

Figure S3. ^{13}C NMR spectra of PEGMEA and P(PEGMEA) from the 3D composite. Insets show the corresponding structural formula of PEGMEA and P(PEGMEA). The solvents are deuterated N,N-dimethylformamide.

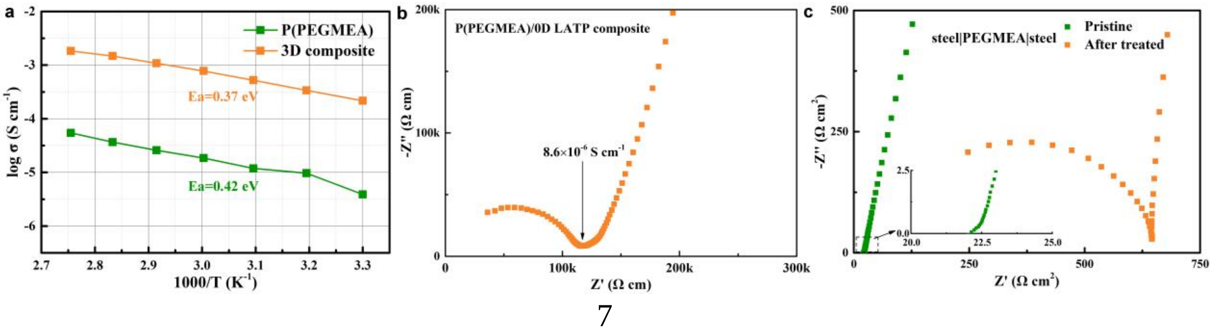

Figure S4. (**a**) Relation between ionic conductivity of electrolyte and temperature for P(PEGMEA) and 3D composite. EIS plots of steel|steel symmetrical cells based on (**b**) P(PEGMEA)/0D LATP composite, (**c**) pristine and after thermally treated steel|PEGMEA|steel symmetrical cells.

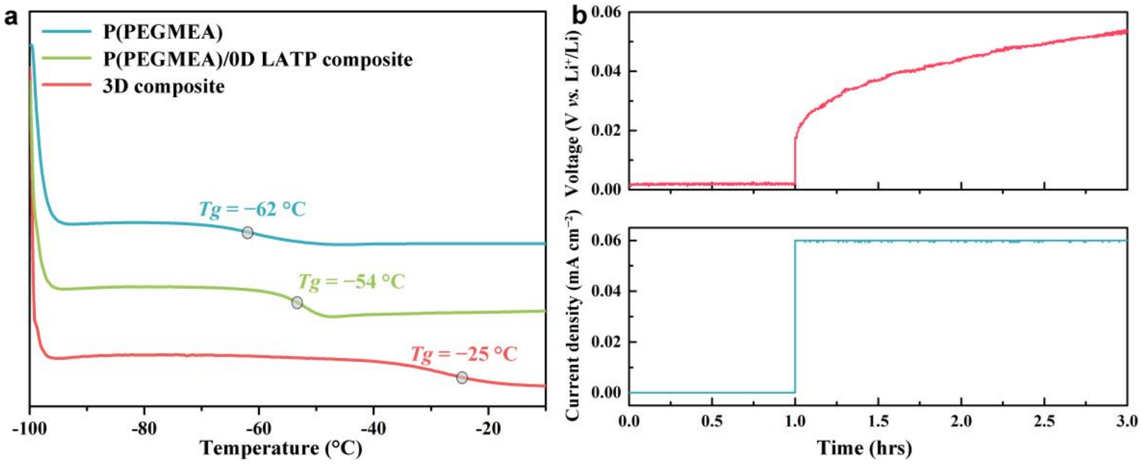

Figure S5. (**a**) DSC profiles of P(PEGMEA), P(PEGMEA)/0D LATP composite and the 3D composite. (**b**) D.C. galvanostatic cycle of ^{6}Li|3D composite|^{6}Li symmetrical cell at room temperature. The area specific capacity is 0.18 mA h cm^{-2} at a current density of 0.06 mA cm^{-2}.

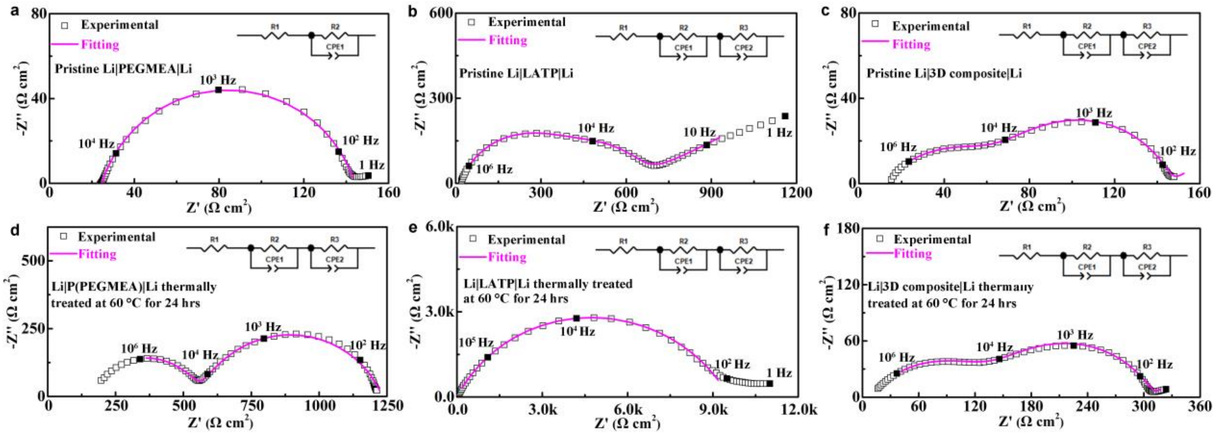

Figure S6. EIS plots and equivalent circuit models of pristine and after thermally treated Li−Li symmetrical cells based on (**a**) and (**d**) PEGMEA, (**b**) and (**e**) LATP, (**c**) and (**f**) 3D composite. The characteristic frequencies are marked.

Figure S6 shows the equivalent circuit models for pristine Li|PEGMEA|Li, Li|LATP|Li, Li|3D composite|Li and after treated Li|P(PEGMEA)|Li, Li|LATP|Li, Li|3D composite|Li symmetric cells based on the corresponding experimental EIS plots. For Li|PEGMEA|Li, R1 in the equivalent circuit represents the Ohmic resistance while R2 represents the interfacial resistance with the capacitance of ~ 10^{-5} F. After thermally treated at 60 °C for 24 hrs, the

span of the first depressed semi-circle, R1 and R2 represents the Ohmic resistance of P(PEGMEA), while the span of the second semi-circle, R3 represents the interfacial resistance with the corresponding capacitance of ~ 10^{-6} F. For pristine Li|LATP|Li symmetrical cell, R2 represents the Ohmic resistance while R3 represents the interfacial resistance with the capacitance of ~ 10^{-7} F. After stored at 60 °C for 24 hrs, R2 represents the Ohmic resistance while R3 represents the interfacial resistance with the capacitance of ~ 10^{-8} F. For pristine Li|3D composite|Li, R1 and R2 in the equivalent circuit represents the Ohmic resistance while R3 represents the interfacial resistance with the capacitance of ~ 10^{-6} F. After thermally treated at 60 °C for 24 hrs, the span of the first depressed semi-circle, R2 represents the Ohmic resistance of 3D composite, while the span of the second semi-circle, R3 represents the interfacial resistance with the corresponding capacitance of ~ 10^{-6} F. The fitting results are summarized in Table S1.

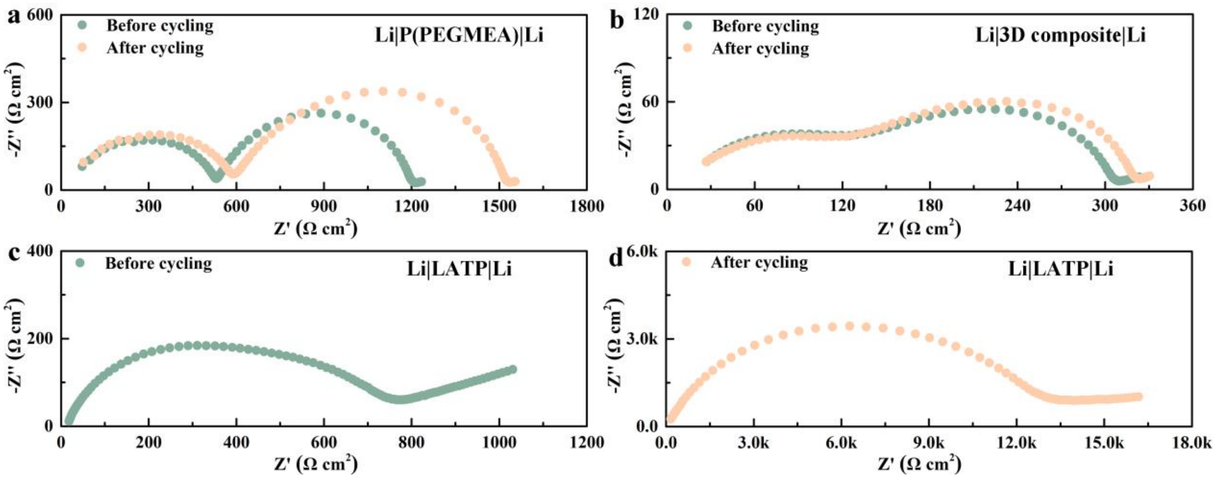

Figure S7. The EIS curves of Li|Li symmetric cells based on (**a**) P(PEGMEA), (**b**) 3D composite, and (**c, d**) LATP before and after cycling.

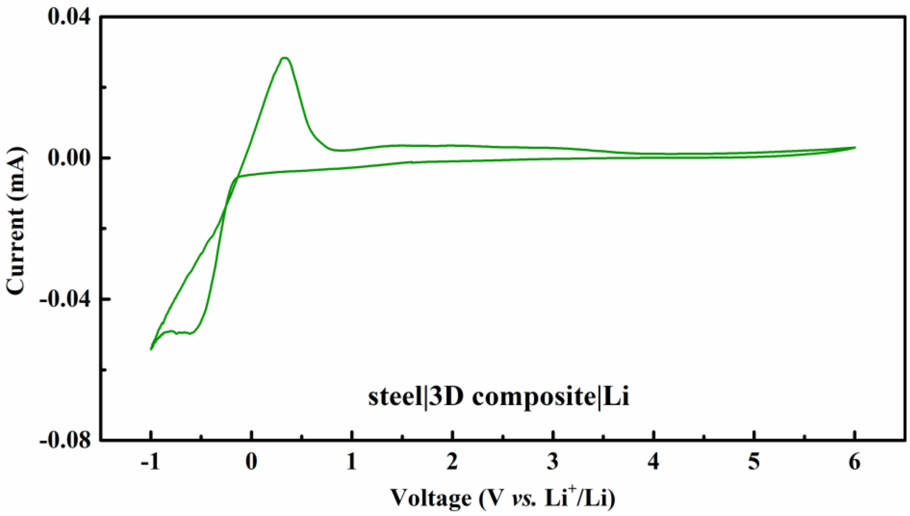

Figure S8. Cyclic voltammetry curve of the steel|3D composite|Li cell.

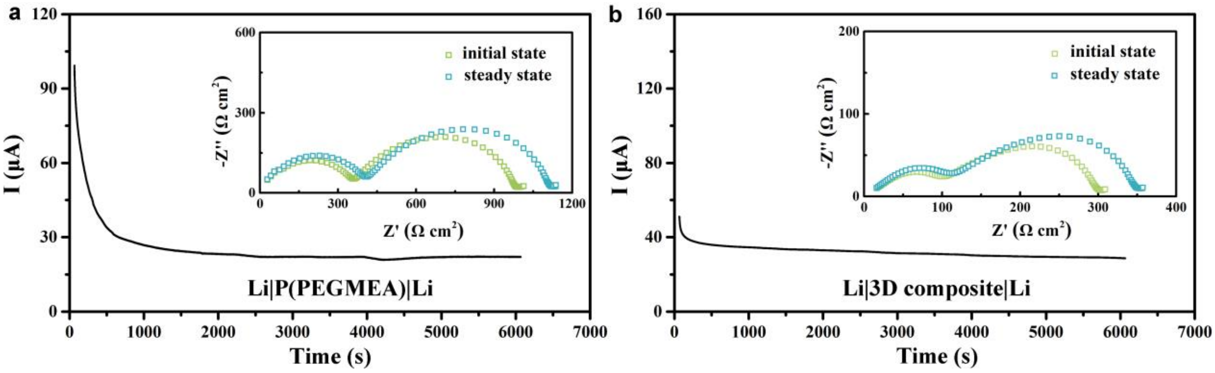

Figure S9. Current variation with time during polarization of (**a**) Li|P(PEGMEA)|Li and (**b**) Li|3D composite|Li symmetrical cell at room temperature.

The electrochemical window and Li^+ transference number (t_{Li^+}) are other two keys to one outstanding electrolyte. Figure S8 shows that the electrochemical window of the 3D composite is over 4.5 V *vs.* Li^+/Li. Higher decomposition voltage suggests the 3D composite is suitable for higher voltage cathode to promote the energy density of SSLB. To reduce the polarization and suppress the formation of lithium dendrites, a high t_{Li^+} is critical. Via a galvanostatic polarization method (Figure S9), t_{Li^+} of the 3D composite is estimated to be 0.48, 4 times higher than that of the P(PEGMEA), 0.12. The high t_{Li^+} may be attributed to the large fraction of LATP (46 vol.%) with a t_{Li^+} of 1. The high room temperature ionic conductivity, wide electrochemical window, and high t_{Li^+} suggest that the 3D composite can

be one promising electrolyte candidate for SSLB.

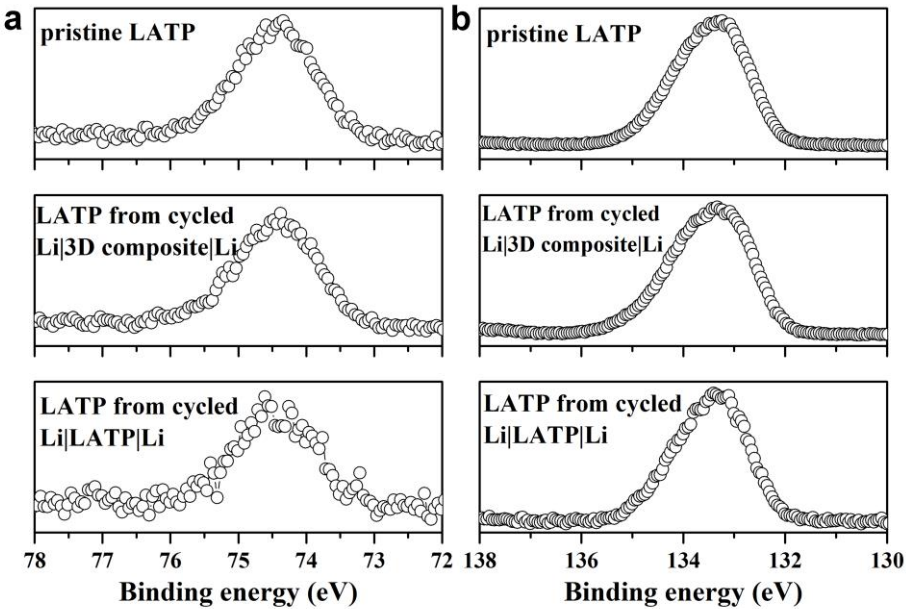

Figure S10. Al 2p (**a**) and P 2p (**b**) XPS spectra of the pristine LATP and LATP after cycling in the Li|3D composite|Li and Li|LATP|Li symmetric batteries.

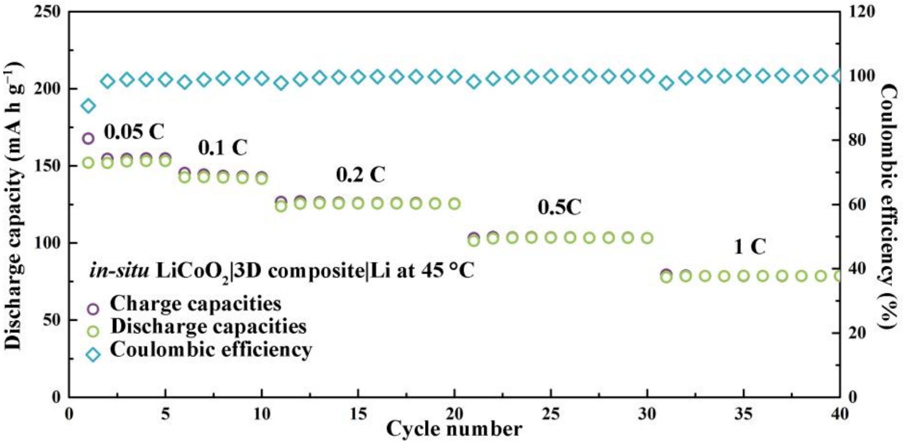

Figure S11. Performance of *in-situ* $LiCoO_2$|3D composite|Li SSLB at different rates.

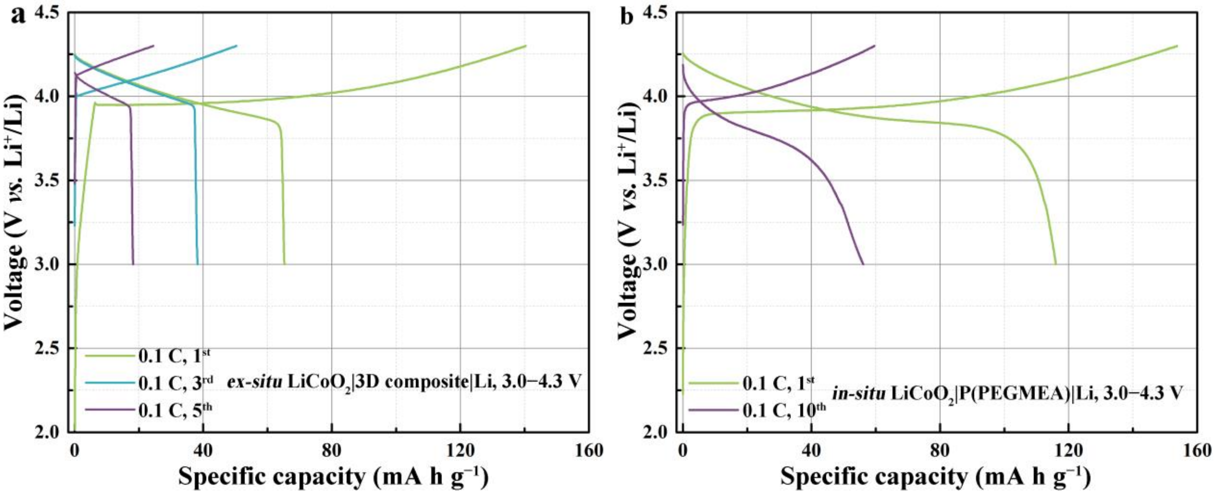

Figure S12. Charge-discharge curves of (**a**) *ex-situ* $LiCoO_2$|3D composite|Li and (**b**) *in-situ*

$LiCoO_2$|P(PEGMEA) |Li SSLBs at 45 °C.

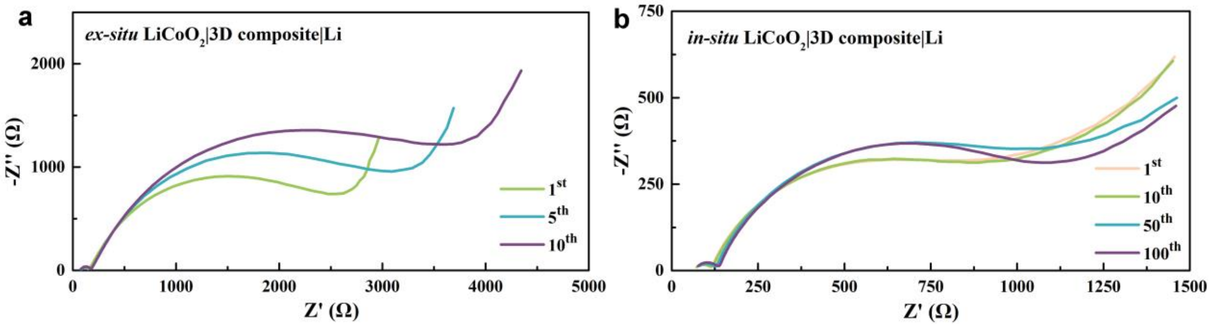

Figure S13. Impedance evolution of the (**a**) *ex-situ* and (**b**) *in-situ* $LiCoO_2$|3D composite|Li SSLBs with cycling.

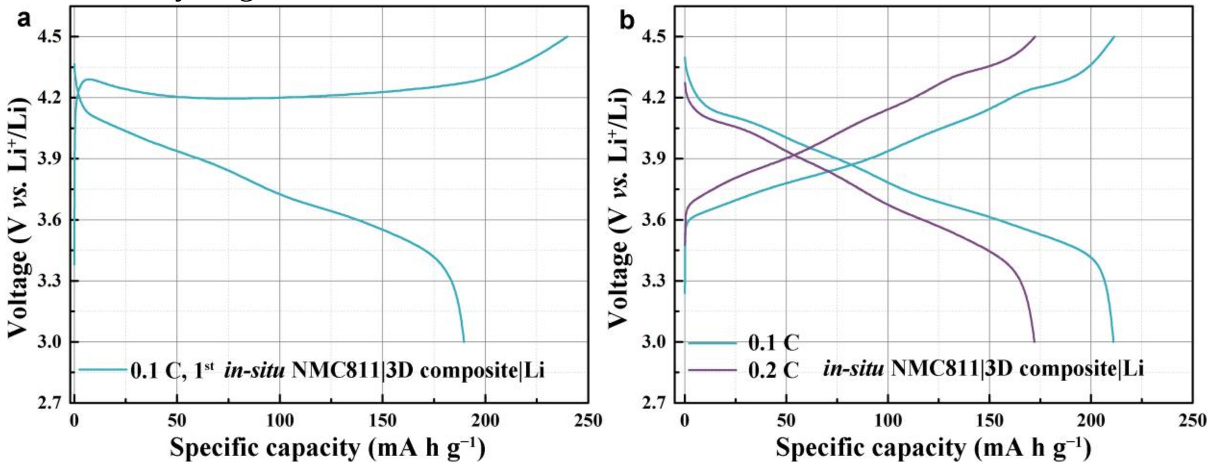

Figure S14. (**a**) Initial charge-discharge curve of *in-situ* NMC811|3D composite|Li SSLB at 3.0 − 4.5 V *vs.* Li^+/Li at room temperature. (**b**) Charge-discharge curves of *in-situ* NMC811|3D composite|Li SSLB at 3.0 − 4.5 V vs. Li^+/Li at different current density.
